# Serbia within the European context: An analysis of premature mortality

**DOI:** 10.1186/1478-7954-7-12

**Published:** 2009-08-05

**Authors:** Milena Santric Milicevic, Vesna Bjegovic, Zorica Terzic, Dejana Vukovic, Nikola Kocev, Jelena Marinkovic, Vladimir Vasic

**Affiliations:** 1 Institute of Social Medicine, School of Medicine University of Belgrade, Dr Subotica 15, 11000 Belgrade, Serbia; 2 Institute of Medical Statistics and Informatics, School of Medicine University of Belgrade, Dr Subotica 15, 11000 Belgrade, Serbia; 3 Department of Statistics and Mathematics, Faculty of Economics, University of Belgrade, Kamenicka 6, 11000 Belgrade, Serbia

## Abstract

**Background:**

Based on the global predictions majority of deaths will be collectively caused by cancer, cardiovascular diseases, and traffic accidents over the coming 25 years. In planning future national health policy actions, inter – regional assessments play an important role. The purpose of the study was to analyze similarities and differences in premature mortality between Serbia, EURO A, EURO B, and EURO C regions in 2000.

**Methods:**

Mortality and premature mortality patterns were analysed according to cause of death, by gender and seven age intervals. The study results are presented in relative (%) and absolute terms (age-specific and age-standardized death rates per 100,000 population, and age-standardized rates of years of life lost – YLL per 1,000). Direct standardization of rates was undertaken using the standard population of Europe. The inter-regional comparison was based on a calculation of differences in YLL structures and with a ratio of age-standardized YLL rates per 1,000. A multivariate generalized linear model was used to explore mortality of Serbia and Europe sub-regions with *ln *age-specific death rates. The dissimilarity was achieved with a p ≤ 0.05.

**Results:**

According to the mortality pattern, Serbia was similar to EURO B, but with a lower average YLL per death case. YLL patterns indicated similarities between Serbia and EURO A, while SRR YLL had similarities between Serbia and EURO B. Compared to all Europe sub-regions, Serbia had a major excess of premature mortality in neoplasms and diabetes mellitus. Serbia had lost more years of life than EURO A due to cardiovascular, genitourinary diseases, and intentional injuries. Yet, Serbia was not as burdened with communicable diseases and injuries as were EURO B and EURO C.

**Conclusion:**

With a premature mortality pattern, Serbia is placed in the middle position of the Europe triangle. The main excess of YLL in Serbia was due to cardiovascular, malignant diseases, and diabetes mellitus. The results may be used for assessment of unacceptable social risks resulting from health inequalities. Within intentions to reduce an unfavourable premature mortality gap, it is necessary to reconsider certain local polices and practices as well as financial and human resources incorporated in the prevention of disease and injury burden.

## Background

The start of the new millennium was an appropriate time for the examination and reform of current health policies and practices for many countries worldwide. In addition to financial crises, health inequalities marked the beginning of the century. Based on the updated Global Burden of Disease (GBD) Study in 2004, predictions are that > 50% of all deaths will be caused collectively by cancer, cardiovascular diseases, and traffic accidents over the coming 25 years [1]. Other alerts involved divergent trends in global mortality and a slowing of improvements in life expectancy in Europe [1]. In addition, common drivers for changes in actual policies and practices have modified users' expectations and dissatisfaction with health system performance. Recent actions of public health policy professionals included setting the millennium development goals and intensification of activities for strengthening health system performance nationally and internationally [2-5].

Due to awareness of the changing settings and related financial flows, it is urgent to objectively assess national health patterns [6]. The GBD study has assisted in the balanced evaluation of health problems worldwide [7]. In fact, the GBD study is a source for accurate information on diseases, injuries, and risk factors for premature mortality, morbidity, and disability [7]. Moreover, such scientific methodology provides a specific look at the operation of a country's health system and its supporting mechanisms.

Serbia is a small country in southeast Europe; it belongs to the EURO B mortality stratum with respect to the low child and adult mortality rate [8]. After a long period of political, social, and economic instability that started with a violent split between six Yugoslav republics (including Serbia), the public health sector underwent reform [9]. The changes coincided with an economic transition from centrally-planned to market-driven. In accordance with the national orientation to enhance cross-border collaboration, the changes appeared useful to explore Serbia's mortality burden within the European context.

Former studies on the burden of diseases and injuries in Serbia aimed to introduce professionals with contemporary methodology, to include premature mortality measures in setting national health priorities, and to assess years of life lost (YLL) patterns within country regions [10-12]. The Serbian Burden of Disease Study [13] was conducted to estimate national disability adjusted life years due to selected diseases, injuries, and risk factors. In planning future national health policy actions, inter-regional assessments play an important role [14,15] and are even more instructive in circumstances of scarce financing. We aimed to explore beneficial directions for health improvement in Serbia.

The purpose of the study was to analyze the similarities and differences in premature mortality between Serbia, EURO A, EURO B, and EURO C regions in 2000.

## Methods

The study populations were Serbia, EURO A, EURO B, and EURO C in 2000. The population of Serbia was 7,550,855 (49% male and 51% female) [16]. The population was estimated with backward projection from the 2002 Census and without a migration component, but Kosovo and Metohija were excluded due to the unavailability of data. The Serbian electronic mortality database was obtained from the Republic's Statistical Office [16]. Data on the population, mortality, and YLL of EURO A, EURO B, and EURO C were obtained from the GBD 2000 estimates report [8,17,18].

The completeness of the Serbian mortality database was 98% in 2000 [13]. The issuing and distribution of death certificates in the state is coordinated between the Ministry of Internal Affairs and the Ministry of Health [19-22]. All deaths occurring in the territory of the Republic of Serbia are registered with a completed death file (certificate of death and statistical paper DEM-2 form) [19,20]. The procedure is consistent throughout the country and comprises several levels of control and verification [21,22]. A death certificate can be obtained by an authorized physician in the health care organization, a coroner, or a forensic doctor. A death file contains information on the cause of death (coded with ICD-10), time and place of death, clinical or forensic postmortem data, and other personal data. The local registrar controls and forwards death files to the referral public health institute within five days, where another trained medical doctor or specialist controls and corrects the file with Death Certificate software within thirty days [21,22]. Authorized statistical bodies compile regional and state unit records and annually report to the public, but with a two-year lag. All the data files are confidential and considered officially undisclosed information.

For this study, mortality data with no personal identification were classified into a comprehensive list of three broad GBD groups, 21 categories of diseases and injuries, and 135 specific conditions, following the structure of the GBD list of conditions and codes [7]. The GBD methodology was used for handling ill-defined codes or missing age information. Therefore, 0.1% deaths with missing age were redistributed proportionally by gender and cause. A total of 8.7% deaths with ill-defined conditions (U161) were redistributed proportionally by age and gender, in the age group < 5 years – across communicable diseases, maternal, perinatal, and nutritional conditions (U001); and in age groups ≥ 5 years – within non-communicable diseases (U059). Then, 3.4% of deaths assigned with code U077 of malignant neoplasms were proportionally redistributed by age and gender in all specified sites, and 0.7% of ill-defined injury deaths (U162) – across all unintentional injuries (U149). Also, 11% of cardiovascular diseases coded U110 were redistributed to ischemic heart disease in accordance with the GBD regression formula [7].

In this paper, we analyzed diseases and injuries that were responsible for approximately > 99% of male and female mortality in Serbia in 2000. Disease categories that contributed < 0.5% to overall mortality in both genders were not presented in this analysis. Apart from the three broad GBD groups: communicable, maternal, perinatal, and nutritional conditions (U001), non-communicable diseases (U059), and injuries (U148), this study included 12 major disease categories: infectious and parasitic diseases (U002), respiratory infections (U038), perinatal conditions (U049), malignant neoplasms (U060), diabetes mellitus (U079), neuropsychiatric conditions (U081), cardiovascular diseases (U104), respiratory diseases (U111), digestive diseases (U115), genitourinary diseases (U120), unintentional injuries (U149), and intentional injuries (U156).

Mortality and premature mortality patterns were analyzed according to cause of death by gender and seven age intervals (0 – 4, 5 – 14, 15 – 24, 25 – 44, 45 – 54, 55 – 69, and ≥ 70 years). Consistent with the GBD Study methodology, YLL were calculated as the number of years lost from deaths occurring before the maximum attainable life expectancy at birth, with a 3% time discount rate for YLL in the future and age-weighting [7]. To make possible the comparison with the burden of disease in other countries, we applied the GBD Study life expectancies at birth: 82.5 years for females (the Coale and Demeny model life table for West level 26) and 80 years for males (standard model life table, the Second edition of West level 25, available in the United Nations Population Division software package MORTPAK) [23,24]. The time discount rate stands for the net present value of a healthy life year gained in 10 years time and is worth 24% less than one gained now. In the absence of national age weights, we applied the GBD Study weights representing broad social preference in which a year of healthy life lived at young and older ages is weighted less than for other ages [7].

The study results are presented in relative (%) and absolute terms (age-specific and age-standardized death rates per 100,000 population, and age-standardized YLL rates per 1,000). Direct standardization of rates was undertaken using the standard population of Europe [25]. A multivariate generalized linear model (GLM) was used to explore mortality of Serbia and European sub-regions due to broad GBD groups with *ln *age-specific death rates. The dissimilarity was achieved with a p ≤ 0.05.

The inter-regional comparison of premature mortality patterns with respect to age, gender, and cause of death distribution was based on a calculation of differences in YLL structures (Serbian YLL structure minus European sub-regional YLL structure) and with a ratio of age-standardized YLL rates per 1,000 – SRR YLL (where Serbia YLL rates were the denominator).

The data analysis and graphic presentation were done in MS Office 2007, while GLM was performed with SPSS for Windows, version 15.0.

## Results

### Mortality patterns

In 2000 there were 104,042 deaths (52% in males and 48% in females) in Serbia. Non-communicable diseases accounted for a large proportion of deaths in males and females in Serbia (92% and 94%, respectively), EURO A (88% and 90%, respectively), EURO B (82% and 88%, respectively), and EURO C (75% and 92%, respectively). Cardiovascular diseases and malignant neoplasms were collectively responsible for > 60% of total mortality, while other causes had minor shares (Table 1; see Additional file 1).

**Table 1:** Total number of deaths and YLL by disease categories: Serbia and European sub-regions^1 ^in 2000

Selected disease categories (GBD Code)	Serbia^2^		EURO A^3^		EURO B^3^		EURO C^3^	
	
	Deaths	YLL	Deaths	YLL	Deaths	YLL	Deaths	YLL
*All Causes (U000)*	104042	814023	3986734	24749640	1868999	20493250	3604321	37067216
*Communicable, maternal, perinatal and nutritional conditions (U001)*	2350	36721	237564	1497486	174837	4571939	144426	3011811
Infectious and parasitic diseases (U002)	700	9432	49196	483596	62031	1670130	79502	1664087
Respiratory infections (U038)	1062	8729	169663	606236	67345	1447835	43875	688149
Perinatal conditions (U049)	543	18000	11428	378876	40071	1329618	18072	599816
*Non-communicable diseases (U059)*	97571	710402	3553238	20339741	1579829	13352506	2991428	24309564
Malignant neoplasms (U060)	19607	193978	1058472	7866460	281188	3032936	504448	5154102
Diabetes mellitus (U079)	2731	20723	89452	446206	28734	264771	21071	264771
Neuropsychiatric conditions (U081)	1362	16162	178278	1146462	24278	457106	37606	710146
Cardiovascular diseases (U104)	64013	393580	1673460	7434356	1051406	7108333	2130728	14636178
Respiratory diseases (U111)	4067	27913	213028	994519	72339	673706	122340	973063
Digestive diseases (U115)	3313	30726	183448	1346282	75004	943173	118772	1553977
Genitourinary diseases (U120)	1722	13732	60455	253777	25139	316520	26398	329485
*Injuries (U148)*	4121	66899	195931	2912413	114333	2568806	468468	9745841
Unintentional injuries (U149)	1964	34757	140491	1921897	79513	1778892	298377	6137215
Intentional injuries (U156)	2159	32140	55440	990516	34820	789913	170090	3608626
*Other*	799	14151	103922	880457	27131	680316	33044	747601

^1^EURO A constitute: Andorra, Austria, Belgium, Croatia, Cyprus, Czech Republic, Denmark, Finland, France, Germany, Greece, Iceland, Ireland, Israel, Italy, Luxembourg, Malta, Monaco, Netherlands, Norway, Portugal, San Marino, Slovenia, Spain, Sweden, Switzerland; EURO B: Albania, Armenia, Azerbaijan, Bosnia and Herzegovina, Bulgaria, Georgia, Kyrgyzstan, Poland, Romania, Serbia and Montenegro, Slovakia, Tajikistan, The FYR Macedonia, Turkey, Turkmenistan, Uzbekistan; EURO C: Belarus, Estonia, Hungary, Kazakhstan, Latvia, Lithuania, Republic of Moldova, Russian Federation, Ukraine [8];

^2^Source: Serbian Burden of Disease Study [13];

^3^Sources: Global Burden of Disease (GBD) 2000: version 3 estimates [17,18].

**Figure 1:**
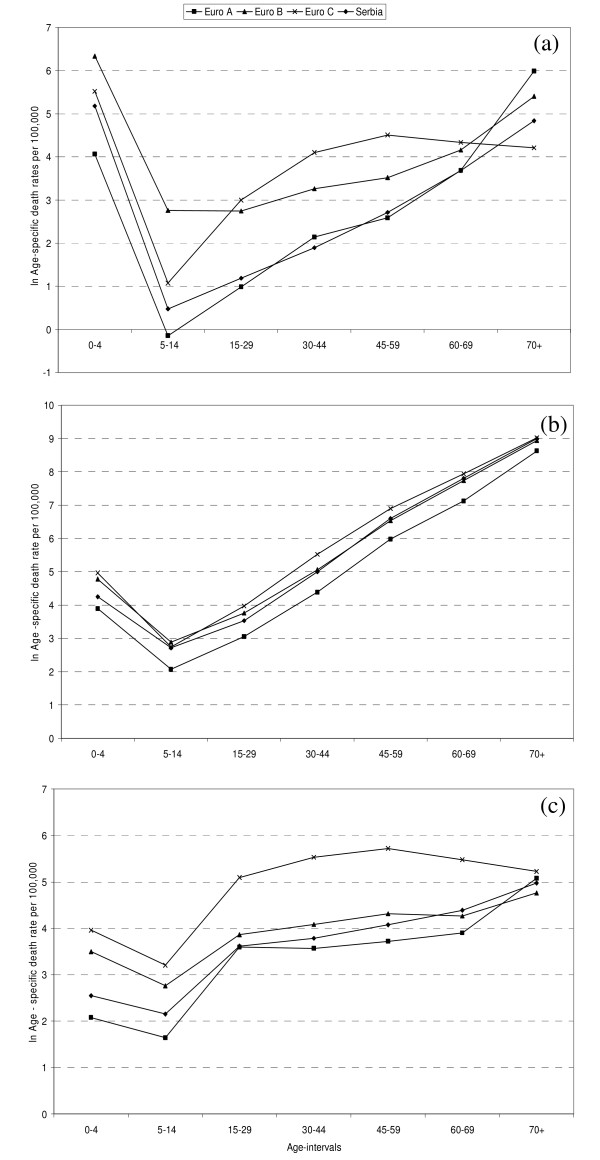
**Log-plots of age-specific death rates (per 100,000) by communicable, maternal, perinatal and nutritional conditions (a); non-communicable diseases (b); and injuries (c), in Serbia and Europe in 2000**.

According to age-specific death rates, Serbia was significantly different from EURO B (p < 0.005) and EURO C (p < 0.005) for communicable, maternal, perinatal, and nutritional conditions (Figure 1a); it had higher age-specific death rates than EURO A (p < 0.005) for non-communicable diseases (Figure 1b), and was dissimilar statistically from Euro C (p < 0.002) with respect to injuries (Figure 1c).

For standardized death rates, Serbia was alike EURO A for communicable, maternal, perinatal, and nutritional conditions, and similar to EURO B for non-communicable diseases, but it had four times lower standardized death rate of injuries than EURO C (see Additional file 2). The four major cause categories were cardiovascular diseases, malignant neoplasms, respiratory, and digestive diseases. Additional causes were diabetes mellitus in Serbia, neuropsychiatric conditions in EURO A, and unintentional injuries in EURO B and EURO C. Males had higher standardized death rates than females due to all causes, with the exception of diabetes mellitus for which the rates for both sexes were similar (see Additional file 2).

In general, the standardized death rates were higher, particularly at ≥ 45 years in all regions. However, malignant neoplasms start increasing at ≥ 30 years of age in males and females of Serbia and EURO C, as do cardiovascular diseases in males of all populations, except EURO A, and in females of EURO C (see Additional file 2).

### Premature mortality patterns

**Figure 2:**
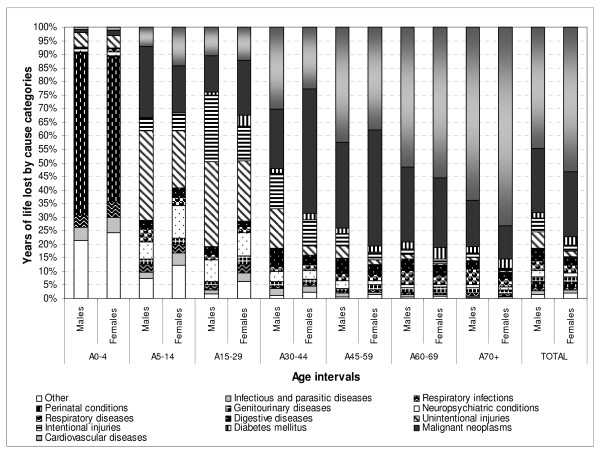
**Distribution of selected causes of YLL (%) by age intervals and gender in Serbia in 2000**.

**Figure 3:**
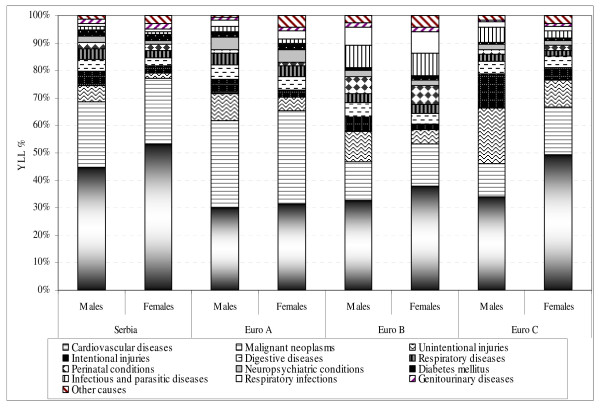
**YLL (%) by gender, causes, and regions in 2000**.

In Serbia, more than 800,000 total YLL were estimated, out of which males had lost 31% more years of life than females (Figure 2). The selected main disease categories caused almost all of the YLL in Serbia (99%), EURO A (96%), EURO B (97%), and EURO C (98%; Figure 3). Most YLL in Serbia were due to cardiovascular diseases (48%) and malignant neoplasms (24%) (Table 1).

The estimated average YLL per death for both males and females in Serbia (9 and 7 YLL, respectively) was lower than in EURO C (13 and 8 YLL, respectively) and EURO B (12 and 10 YLL, respectively). On average, perinatal conditions caused 33 YLL per death in both males and females in all regions, while malignant neoplasms caused 11 YLL in EURO B, 10 YLL in Serbia and EURO C, and 7 YLL in EURO A. Male vs. female average YLL due to cardiovascular diseases was 7 vs. 6 in Serbia, 6 vs. 3 in EURO A, 8 vs. 6 in EURO B, and 9 vs. 5 in EURO C.

Prior to 45 years of age, the population had already lost 19% of total YLL in Serbia, 23% in EURO A, 38% in EURO C, and 48% in EURO B. The main causes for this effect were injuries, neuropsychiatric conditions, infectious and parasitic diseases, respiratory infections, and perinatal conditions. Malignant neoplasms and cardiovascular diseases affected people aged 45 years and older.

**Figure 4:**
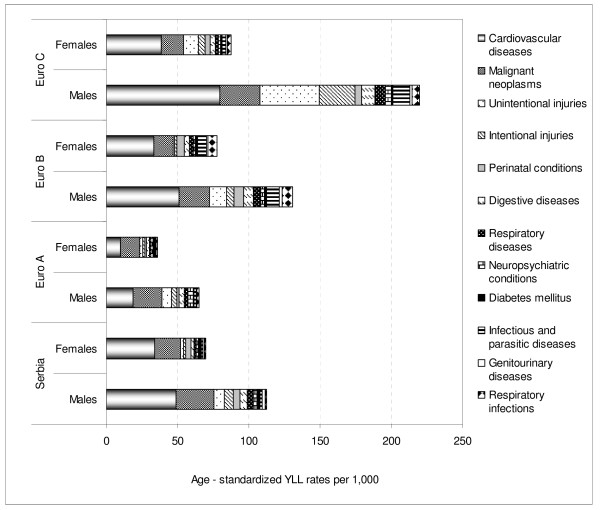
**Age-standardized rates of YLL per 1,000 by gender: Serbia and European sub-regions in 2000**.

In Serbia, total age-standardized YLL rate was 93 per 1,000. Males had higher rates then females (115 vs. 72; see Additional file 3). Serbia had higher rates than EURO A, but lower than EURO C and EURO B for cardiovascular diseases, unintentional, and intentional injuries. Serbia and EURO C had similar rates of malignant neoplasms (Figure 4).

The analysis of premature mortality of Serbia within the European context included a calculation of differences in YLL structure (Figure 3), and ratios of age-standardized YLL rates – SRR YLL (according to data in Additional file 3).

Due to cardiovascular diseases, Serbian males and females had a surplus of YLL in comparison to their counterparts in EURO A (by 16% and 22%, respectively), EURO B (by 12% and 15%, respectively), and EURO C (by 11% and 4%, respectively). Malignant neoplasms caused additional YLL excess in Serbian males and females in comparison to EURO B (by 9.4% and 8.4%, respectively), and EURO C (by 12% and 6.5%, respectively).

With respect to SRR YLL, Serbian males and females resembled EURO B for cardiovascular and genitourinary diseases and EURO C for malignant diseases and perinatal conditions.

For SRR YLL across almost all age groups, there were higher YLL in Serbia than in EURO A in both males and females for cardiovascular diseases (0.4- and 0.3-fold, respectively), malignant neoplasms (0.8- and 0.7-fold, respectively), diabetes mellitus (0.4- and 0.3-fold, respectively), genitourinary diseases (by 0.3-fold in each), intentional injuries (by 0.6-fold in each), and perinatal conditions (0.3- and 0.4-fold, respectively).

With respect to SRR YLL, there were higher YLL in Serbia than in EURO B in both males and females for malignant neoplasms (by 0.8-fold in each), diabetes mellitus (by 0.7-fold in each), and intentional injuries only among those ≥ 45 years of age (in total, 0.9- and 0.8-fold, respectively).

With respect to SRR YLL, there were higher YLL in Serbia than in EURO C in both males and females for diabetes mellitus (0.6- and 0.4-fold, respectively), and genitourinary diseases (0.9- and 0.8-fold, respectively).

On the other hand, SRR of YLL indicated a lower burden in Serbia than in EURO B for both males and females regarding infectious and parasitic diseases (5.3- and 6.5-fold, respectively), respiratory infections (5.4- and 6.0-fold, respectively), perinatal conditions (1.4- and 1.6-fold, respectively), unintentional injuries (1.6- and 2.0-fold, respectively), and digestive diseases (1.4- and 1.7-fold, respectively).

Compared to EURO C, Serbia had a lower burden of YLL in both males and females for infectious and parasitic diseases (7.1- and 2.9-fold, respectively), unintentional injuries (5.6- and 5.2-fold, respectively), intentional injuries (4.0- and 2.4-fold, respectively), respiratory infections (3.7- and 2.3-fold, respectively), digestive diseases (by 1.9-fold in each), neuropsychiatric conditions (by 1.5-fold in each), and cardiovascular diseases (1.6- and 1.1-fold, respectively).

## Discussion

This research aimed to explore Serbian premature mortality within the European context as an extension of the national burden of disease study. Differences between Serbia and European sub-regions were identified in terms of excess YLL structures and in terms of SRR YLL for selected diseases, along with age and gender distribution. According to the mortality pattern, Serbia was similar to EURO B, but with a lower average YLL per death case. The burden due to cardiovascular diseases and cancers accounted for approximately two-thirds of the total YLL in Serbia. YLL patterns indicated similarities between Serbia and EURO A, while SRR YLL showed similarities between Serbia and EURO B. Compared to all European sub-regions, Serbia had a major excess of premature mortality due to neoplasms and diabetes mellitus. Serbia had lost more years of life than EURO A due to cardiovascular, genitourinary diseases, and intentional injuries. In addition, in Serbia the major premature mortality burden shifted towards middle life years, in particular among males and due to non-communicable diseases. Yet, Serbia was not as burdened with communicable diseases and injuries as were EURO B and EURO C.

Some of the identified similarities between Serbia and Europe with respect to premature mortality were expected because of the geographical and historical conditions. Geographically, Serbia borders with countries in EURO A, the former Yugoslav republic of Croatia [8]. Also, Serbia is a neighbor of Hungary (EURO C) and the Former Yugoslav Republic of Macedonia (EURO B). Some of the disparities could be explained by social and economic circumstances [6,26,27] and recent developments in Serbia: army conflicts accompanied with waves of internally-displaced persons and refugees, two huge inflations in 1989 and 1993, several years of economic sanctions, and NATO bombing in 1999.

The demographic transition, biological, lifestyle, or cultural differences certainly model the mortality pattern [1]. Similar to the results of our study, males had most YLL in countries with well-established market economies (60%), and in European countries with former socialist economies (62%) [28]. The aging of the Serbian population (the aging index was 1 and the median age around 40 years) [16] contributed to the compression of morbidity and the predominance of non-communicable diseases among adults and the elderly. In addition, the proportion of the youngest in the Serbian population (about 16% in the age interval 0 – 14 years) [16] could contribute to the allocation of burden mainly across the adult population.

This study had some limitations. Although Serbian registration of deaths is considered almost complete and with relatively accurate information on causes of death, 80% completeness of adult male and female mortality registration was calculated in two Serbian regions with the Brass Growth Balance method [12]. Yet, that method is more suitable for stable populations where partial birth and death rates from registered deaths have a linear relationship [29]. Nevertheless, the study findings may be biased due to various sources of mortality data, as well as different diagnostics and coding practices across compared regions. As a precaution, in a following comparative research, any improvements in the quality of data should be accounted for. For example, in Serbia data on ill-defined causes of mortality were almost halved in 2006 (4.8%) compared to 2000 (8.7%) [30]. Advanced work of the study should consider some issues as in other studies [31-34], e.g., major conditions within cause categories, the relationship to ethnicity, residence, employment status or level of education, thus providing a more valuable tool for public health planning.

Notwithstanding the shortcomings, this is the first study that objectively quantified the premature mortality gap between Serbia and the European sub-regions. Therefore, the findings could be used to indicate convergence of current national health plans for some diseases with health plans in Europe. They advocate strategic orientation of country health planners and decision-makers for public health activities within Europe and integration of health protection in other policies at the state level. The results support country participation in intraregional collaboration that will facilitate initiatives for promotion of healthy lifestyles and better disease management and that may improve institutional health care protocols.

Mortality registration needs to be improved at all levels by modernization of control, knowledge, coding practices, and death verification procedures. This is of particular relevance regarding adult mortality under-registration, and for forensic cases, e.g. injuries, since the clinical documentation and death files are among the key elements in juridical procedures.

After several years of slow transition, Serbia should have a new systematic revision of the national burden of disease in order to provide a specific aspect on the health sector reform outcomes and possible improvements within the European context.

## Conclusion

With a premature mortality pattern, Serbia is placed in the middle of the European triangle. The main excess of YLL in Serbia was due to cardiovascular, malignant diseases, and diabetes mellitus. These results may be used for assessment of unacceptable social risks resulting from health inequalities. In order to reduce an unfavourable premature mortality gap, it is necessary to reconsider certain local polices and practices as well as the financial and human resources needed for prevention of disease and injury burden.

## Competing interests

The authors declare that they have no competing interests.

## Authors' contributions

MSM, VB, and ZTS made substantial contributions to the study's conception and design, analysis and interpretation of data, and drafting the manuscript. JM participated in the study's design, coordination, and critical revision for important intellectual content. NK and VV performed data acquisition and statistical analysis. DV participated in drafting the manuscript sequence alignment and edited the language. All authors approved the final manuscript.

## Supplementary Material

Additional file 1 **Total number of deaths and YLL by disease, age, and gender in Serbia in 2000**. Source: [13]. The data provided represent the distribution of the total number of deaths and years of life lost by disease, age, and gender in Serbia in 2000. Click here for file

Additional file 2 **Cause-specific age-standardized death rates per 100,000; Serbia, EURO A, EURO B, and EURO C in 2000 (standard European population)**. The data provided represent the distribution of cause-specific age-standardized death rates per 100,000 (standard European population), in four regions: Serbia, EURO A, EURO B, and EURO C in 2000. Click here for file

Additional file 3 **Cause-specific age-standardized rates of YLL per 1,000; Serbia, EURO A, EURO B, and EURO C in 2000 (standard European population)**. The data provided represent the distribution of cause-specific age-standardized rates of years of life lost per 1,000 (standard European population), in four regions: Serbia, EURO A, EURO B, and EURO C in 2000. Click here for file
